# Repeat abortion and associated factors among women seeking abortion services in northwestern China: a cross-sectional study

**DOI:** 10.1186/s12889-021-11653-4

**Published:** 2021-09-06

**Authors:** Chen Li, Jianmin Gao, Jinlin Liu

**Affiliations:** 1 School of Public Health, Xi’an Jiaotong University Health Science Center, Xi’an, 710061 Shaanxi China; 2 School of Public Policy and Administration, Xi’an Jiaotong University, Xi’an, 710049 Shaanxi China; 3 School of Public Policy and Administration, Northwestern Polytechnical University, Xi’an, 710129 Shaanxi China

**Keywords:** Repeat abortion, Unintended pregnancy, Women, Sexual partner, China

## Abstract

**Background:**

Repeat abortion is a significant public health problem in China. International knowledge about repeat abortion and its associated factors in Chinese women is scarce. This study aimed to analyze the prevalence of repeat abortion among women seeking abortion services with unintended pregnancies in northwestern China and to identify factors associated with the repeat abortion from both two perspectives of abortion seekers themselves and their sexual partners.

**Methods:**

This cross-sectional survey was conducted from May 1st to May 31st, 2020, in 90 medical institutions in Xi’an, the largest city in northwestern China. All women seeking abortions within the first 12 weeks of pregnancy were invited to participate in this survey; however, only those abortion seekers with unintended pregnancies were extracted and included in this study. Pearson’s chi-squared tests, Kolmogorov-Smirnov tests, and binary logistic regression analysis were performed.

**Results:**

Of 3397 abortion seekers, 56.6% (1924) were undergoing repeat abortions. Participants who were older than 30 years (OR: 1.37, 95% CI: 1.08–1.73 for 31–35 years; 1.82, 1.29–2.57 for ≥36 years), received a low-level education (1.86, 1.42–2.43 for ≤senior high school; 1.46, 1.17–1.83 for junior college), were jobless (2.46, 1.18–5.13), had one child (1.54, 1.10–2.17), had a general (1.60, 1.28–1.98) or no (2.51, 2.02–3.11) cognition of possible adverse health effects of having abortions, and had used contraception at the time of conception, i.e., condoms (1.33, 1.09–1.61), withdrawal (1.43, 1.12–1.84), and emergency measures (1.48, 1.09–1.99) were more likely to undergo a repeat abortion. Besides, participants whose sexual partners were older than 30 years (1.33, 1.06–1.68 for 31–35 years; 2.13, 1.56–2.91 for ≥36 years), attained a low-level education (1.66, 1.28–2.15 for ≤senior high school; 1.38, 1.10–1.74 for junior college), received a high-level monthly income (1.34, 1.08–1.65 for ≥6001 Yuan), and had a weak or very weak willingness to use contraception (6.84, 2.42–19.33) were more likely to have a repeat abortion.

**Conclusions:**

The study findings highlight the problem of repeat abortion in China and suggest the need for government and civil society to increase efforts to reduce the risks of unintended pregnancy and repeat abortion in China. One approach may be to offer better access to reproductive health and contraception knowledge to women and their sexual partners and to promote their correct, consistent, and effective contraception practice.

**Supplementary Information:**

The online version contains supplementary material available at 10.1186/s12889-021-11653-4.

## Background

Unintended pregnancy and induced abortion are experienced by women around the world [1]. Between 2015 and 2019, approximately 121 million unintended pregnancies occurred every year, of which 61% ended in abortion, corresponding to 39 abortions per 1000 women aged 15–49 years and a total of 73 million abortions each year [1]. There is a global consensus that abortion is a public health problem that needs substantial attention [2]. Besides, many women experience more than one abortion throughout their reproductive years [3], and repeat abortion has also been noted as a significant and growing public health problem worldwide [2, 3]. The proportion of repeat abortion among all abortions had increased in Sweden (from 19% in 1975 to 38% in 2008), New Zealand (from 23% in 1991 to 38% in 2011), and France (from 18% in 1990 to 28% in 2002 and to 41% in 2011) [2, 3]. In the U.S., the percentage of repeat abortion among women undergoing an abortion ranged from 44.8 to 58.8% [4–7]. Other studies conducted in the U.K., Switzerland, Norway, Italy, Netherlands, Finland, Ethiopia, Georgia, Ghana, Kenya, Nigeria, Tunisia, Vietnam, Nepal, Hungary, Russia, and Canada reported that the prevalence of repeat abortion among women receiving an abortion or having had an abortion were 19.2–23.4% [8, 9], 30.1% [10], 36.7% [11], 60.6% [12], 36.0% [13], 14.1–37.9% [14, 15], 20.3–34.9% [16–18], 69.9% [19], 33.7% [20], 14.3% [21], 23.0% [22], 42.2% [23], 31.7% [24], 32.3% [25], 46.1% [26], 23.6% [27], and 31.8% [28], respectively.

There is evidence that a higher number of previous abortions is associated with an increased risk of adverse perinatal outcomes in a subsequent pregnancy [29–36]. As an indicator of quality of abortion care and reproductive health services for women, repeat abortion could be a pointer to poor linkage between abortion and contraceptive services, and it could also be a pointer to inadequate counseling services linked to abortion care. With these in mind, identifying associated factors is of importance that could help target interventions to those identified as vulnerable to repeat abortions. Currently, many studies have been conducted to identify factors associated with repeat abortion, which included individual sociodemographic characteristics (such as age, education, occupation, income, marriage, residence status, migrant status, and parity), unhealthy behaviors (such as tobacco and alcohol use), contraception patterns (such as contraceptive use at the time of conception or in the period prior to the survey), contraceptive knowledge, and factors related to sexual partners (such as the number of sexual partners and intimate partner violence) [2–21, 23–28, 37–44]. Despite many international publications in this area, there is a lack of consensus regarding all of these factors associated with the repeat abortion.

In China, repeat abortion is a significant and growing public health problem [45, 46]. Induced abortion is legal and is a part of China’s family planning services [47]. With premarital sexual relationships becoming more acceptable in China, the risks of unintended pregnancy and subsequent abortion are increasing accordingly [46, 48]. Furthermore, the universal two-child policy implemented in 2016 may also increase such risks [49]. According to the statistics of the National Health Commission of China, approximately 6.1–9.9 million abortions were performed by the family planning services in China per year from 2000 to 2018 [50]. Meanwhile, a nationwide large-scale survey conducted in 30 Chinese provinces in 2013 reported that the prevalence of repeat abortion among 79,174 women seeking abortions was 64.8% [45], which showed that repeat abortion was highly prevalent in China. Despite several empirical studies on this topic in China, only a few have been published in international journals [49], and most are only available in Chinese and they draw no consistent conclusions on the prevalence and determinants of the repeat abortion among Chinese women. In addition, similar to international studies in other countries, very few studies in China have paid attention to factors related to women’s sexual partners. Given above evidence that repeat abortion is considerable challenge to women’s sexual and reproductive health in China, more research needs to be conducted to analyze the repeat abortion among women in China, especially to identify the factors associated with the repeat abortion.

Based on the above, using the data from a month-long cross-sectional survey among women seeking abortions in Xi’an, the largest city in northwestern China, this study aimed to analyze the prevalence of repeat abortion among these abortion seekers and to identify associated factors from the perspectives of both women and their sexual partners.

## Methods

### Study design and participants

A cross-sectional study was conducted in Xi’an, which is the capital of Shaanxi Province and located in northwestern China. All types of medical institutions, i.e., public or private, general or specialized, and primary, secondary, or tertiary, that can provide abortion services in Xi’an were invited to participate in the study. A total of 90 medical institutions were finally involved (*see* Table 1 *in* Additional file 1), of which 71.1% (64) were public hospitals, 88.9% (80) were general hospitals, and 32.2% (29) and 62.2% (56) were tertiary and secondary hospitals, respectively.

Besides, with reference to the study design in previous studies [45, 46, 48], we applied the convenience sampling strategy to select the study participants. Data were collected consecutively from all women seeking a surgical abortion or a medical abortion within the first 12 weeks of pregnancy in above 90 medical institutions during a study period of 1 month. With the assistance of nurses in each medical institution, an invitation to participate was sent to abortion seekers before they left the medical institutions after receiving the abortion services. However, after the data were cleaned, only those participants who had unintended pregnancies and reported valid data with respect to first versus repeat abortion were included in this study. We used abortion seekers’ answers to one question in the questionnaire to distinguish between women having unintended pregnancies and those with wanted pregnancies but who required an abortion due to medical reasons (*see item 3.2 in* Additional file 2).

We followed the STROBE (strengthening the reporting of observational studies in epidemiology) guidelines when reporting the results of this study [51].

### Data collection and variable measurement

Data were collected between May 1st and May 31st, 2020, at each of the participating medical institutions. By referring to previous relevant studies in China [45, 48] and considering the objectives of this study, a structured questionnaire was developed by the research team and used for data collection (*see* Additional file 2). The questionnaire was anonymous and completed by the abortion seekers themselves. Before the formal survey, we conducted a small-scale pre-survey in two medical institutions to validate, revise, and finalize the questionnaire. All respondents provided verbal consent to participate in the survey.

The questionnaire consisted of three sections. The first section was participants’ sociodemographic characteristics measured by eight variables, which were: (1) age as a continuous variable initially and divided into three groups, i.e., ≤30 years, 31–35 years, and ≥ 36 years; (2) education with three groups, i.e., senior high school or below, junior college, and bachelor’s degree or above; (3) residence status with two groups, i.e., rural and urban; (4) migrant status with two groups, i.e., migrant and nonmigrant; (5) occupation with seven groups, i.e., student, housework, farmer, self-employed, enterprise employee, civil servant/teacher/researcher, and jobless; (6) income per month as a continuous variable initially and divided into three groups, i.e., ≤2500 Yuan, 2501–4000 Yuan, and ≥ 4001 Yuan; (7) marital status with two groups, i.e., unmarried and married; (8) parity with three groups, i.e., no children, 1 child, and ≥ 2 children.

The second section covered the sociodemographic characteristics of the participants’ sexual partners. They included six variables that were age, education, residence status, migrant status, occupation, and income per month, and these variables were set consistently with those of the participants in the first section except for income per month. Income per month of the participants’ sexual partners was a continuous variable initially and then it was divided into three groups, i.e., ≤4500 Yuan, 4501–6000 Yuan, and ≥ 6001 Yuan.

The third part was related to induced abortion and contraceptive use measured by five variables, which included the following: (1) repeat abortion with two groups, i.e., no and yes; (2) contraceptive use at the time of conception with six groups, i.e., nonuse, condom, rhythm, withdrawal, emergency, and other measures such as the combined oral contraceptive pill and implants; (3) contraceptive use during 6 months preceding the survey with six groups (available for multiple choices), i.e., nonuse, condom, rhythm, withdrawal, emergency, and other measures; (4) cognition of the possible adverse health effects of having an abortion with three groups, i.e., know well, general, and don’t know; (5) sexual partners’ willingness to use contraception with five groups: very weak, weak, general, strong, and very strong.

### Data analysis

Data were entered, cleaned, and analyzed using Stata 14.1 (StataCorp, Texas, USA) for MAC. All categorical variables are displayed as counts and percentages. Continuous variables, i.e., participants’ and their sexual partners’ age and income per month, were tested for normality first using the one-sample K-S (Kolmogorov-Smirnov) tests. All *p*-values for these four variables were < 0.001, which indicated a non-normal distribution, and we described them using the “median” and “interquartile range (IQR)”.

Pearson’s chi-squared tests were applied to assess differences in the proportions of the sociodemographic characteristics of the participants and their sexual partners, participants’ cognition of the possible adverse health effects of having an abortion, and contraceptive use between participants undergoing a first abortion and those receiving a repeat abortion. Two-sample K-S tests were also performed to assess differences in the distribution of age and income per month of participants and their sexual partners between participants receiving a first abortion and those with a repeat abortion. *P*-values are displayed.

Multivariate binary logistic regression analysis was performed to determine the factors associated with repeat abortion of the participants. In the regression model, the repeat abortion of participants was set as the dependent variable, and other variables, including sociodemographic characteristics of the participants and their sexual partners, contraceptive use, participants’ cognition of possible adverse health effects of having an abortion, and participants’ sexual partners’ willingness to use contraception, were set as independent variables. However, only those that were already significant in prior univariate analyses, i.e., Pearson’s chi-squared tests and two-sample K-S tests, were included in the logistic regression model. The β (regression coefficient), S.E. (standard error), odds ratio (OR), 95% CI (confidence interval), and *p*-value were reported. A *p*-value < 0.05 was considered to be significant in this study.

## Results

### Study participants

**Fig. 1:**
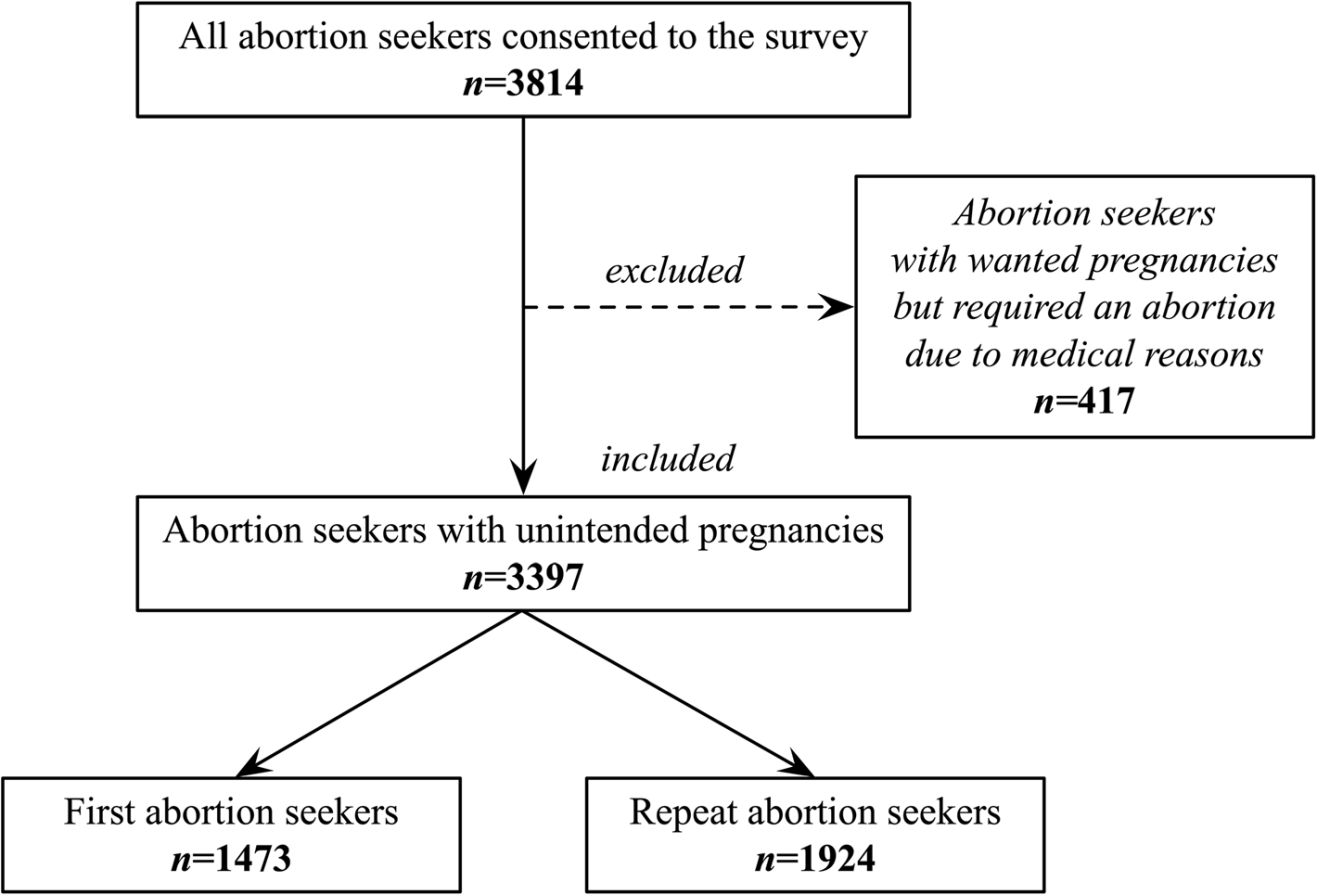
Study profile

Figure 1 shows the profile of the participants. A total of 3814 women seeking an induced abortion in 90 medical institutions participated in the survey, of whom 57.9 and 37.4% attended tertiary and secondary hospitals, respectively, 80.0% attended public hospitals, and 78.6% attended general hospitals (*see* Table 2 *in* Additional file 1).

However, among all abortion seekers in the survey, our study only focused on those abortion seekers with unintended pregnancies, not those with wanted pregnancies but who required an abortion due to medical reasons. As Fig. 1 shows, of all 3814 abortion seekers, 417 were excluded as they were planning to become pregnant but needed an abortion because of medical reasons, and thus 3397 participants were extracted from the original dataset and included in this study. Among these 3397 abortion seekers with unintended pregnancies, 1924 (56.6%) were undergoing a repeat abortion.

### Sociodemographic characteristics

Table 1 shows the sociodemographic characteristics of the participants. Compared with participants undergoing their first abortions, those with a repeat abortion presented significantly higher proportions in the following groups: ≥31 years; receiving an education of senior high school or below; urban resident; nonmigrant; being engaged in occupations of housework, farmer, self-employed, and jobless; married; and having one or more children.

**Table 1:** Sociodemographic characteristics of participants

Characteristics	***N*** (%) or Median (IQR)			***P***-value
	Total	First abortion	Repeat abortion	
**Age** (years)	30 (26–34)	28 (24–32)	31 (28–35)	< 0.001^†^
≤ 30 years	1802 (53.2)	976 (66.3)	826 (43.0)	< 0.001^‡^
31–35 years	974 (28.7)	347 (23.6)	627 (32.7)	
≥ 36 years	614 (18.1)	148 (10.1)	466 (24.3)	
**Education**				< 0.001^‡^
≤ Senior high school	1513 (44.5)	544 (36.9)	969 (50.4)	
Junior college	1070 (31.5)	483 (32.8)	587 (30.5)	
≥ Bachelor’s degree	814 (24.0)	446 (30.3)	368 (19.1)	
**Residence status**				0.014^‡^
Rural	2252 (66.3)	1007 (68.4)	1245 (64.7)	
Urban	1145 (33.7)	466 (31.6)	679 (35.3)	
**Migrant status**				0.002^‡^
Migrant	1183 (34.8)	553 (37.5)	630 (32.7)	
Nonmigrant	2214 (65.2)	920 (62.5)	1294 (67.3)	
**Occupation**				< 0.001^‡^
Student	88 (2.6)	70 (4.8)	18 (0.9)	
Housework	611 (18.0)	238 (16.2)	373 (19.4)	
Farmer	213 (6.3)	88 (6.0)	125 (6.5)	
Self-employed	633 (18.6)	225 (15.3)	408 (21.2)	
Enterprise employee	1300 (38.3)	601 (40.8)	699 (36.3)	
Civil servant etc.	394 (11.6)	192 (13.0)	202 (10.5)	
Jobless	158 (4.7)	59 (4.0)	99 (5.1)	
**Monthly income** (Yuan)	3500 (2385–5000)	3600 (2500–5000)	3500 (2000–5000)	0.120^†^
≤ 2500 Yuan	892 (26.4)	382 (26.0)	510 (26.6)	0.103^‡^
2501–4000 Yuan	1250 (36.9)	570 (38.9)	680 (35.5)	
≥ 4001 Yuan	1243 (36.7)	515 (35.1)	728 (38.0)	
**Marital status**				< 0.001^‡^
Unmarried	841 (24.8)	515 (35.0)	326 (16.9)	
Married	2556 (75.2)	958 (65.0)	1598 (83.1)	
**Parity**				< 0.001^‡^
No children	924 (27.2)	574 (39.0)	350 (18.2)	
1 child	1460 (43.0)	553 (37.5)	907 (47.1)	
≥ 2 children	1013 (29.8)	346 (23.5)	667 (34.7)	

^†^ Two-sample K-S test. ^‡^ Pearson’s chi-squared test

The sociodemographic characteristics of participants’ sexual partners were reported in Table 1 in Additional file 3. In comparison to the sexual partners of participants undergoing their first abortions, the sexual partners of those receiving a repeat abortion presented significantly higher percentages in the following groups: ≥31 years; receiving an education of senior high school or below; being engaged in the occupations of a farmer or self-employed; and receiving a monthly income of 6001 Yuan or more.

### Contraception- and abortion-related characteristics

Table 2 shows participants’ contraceptive use and cognition of the possible adverse health effects of having an abortion and their sexual partners’ willingness to use contraception. Compared with the participants undergoing their first abortions, those with repeat abortions presented a significantly higher proportion in the following groups: having used contraception at the time of conception; having no knowledge of possible adverse health effects of having abortions; and having a weak or very weak willingness to use contraception of participants’ sexual partners.

**Table 2:** Contraceptive use, cognition of possible adverse health effects of having an abortion, and sexual partners’ willingness to use contraception

Characteristics	***N*** (%)			***P***-value
	Total	First abortion	Repeat abortion	
**Contraceptive use at the time of conception**				0.005^†^
Nonuse of contraception	1418 (41.7)	672 (45.6)	746 (38.8)	
Condom	854 (25.1)	348 (23.6)	506 (26.3)	
Rhythm	400 (11.8)	166 (11.3)	234 (12.2)	
Withdrawal	398 (11.7)	157 (10.7)	241 (12.5)	
Emergency	254 (7.5)	100 (6.8)	154 (8.0)	
Other	73 (2.1)	30 (2.0)	43 (2.2)	
**Contraceptive use during six months preceding the survey**				0.646^†^
Nonuse	216 (6.4)	100 (6.8)	116 (6.0)	
One type	2174 (64.0)	935 (63.5)	1239 (64.4)	
Two types or more	1007 (29.6)	438 (29.7)	569 (29.6)	
**Cognition of the possible adverse health effects of having an abortion**				< 0.001^†^
Know well	577 (17.0)	325 (22.1)	252 (13.1)	
General	1250 (36.8)	591 (40.1)	659 (34.3)	
Don’t know	1570 (46.2)	557 (37.8)	1013 (52.7)	
**Sexual partners’ willingness to use contraception**				0.018^†^
Very strong	842 (24.8)	364 (24.7)	478 (24.8)	
Strong	1845 (54.3)	791 (53.7)	1054 (54.8)	
General	678 (20.0)	312 (21.2)	366 (19.0)	
Weak or very weak	32 (0.9)	6 (0.4)	26 (1.4)	

^†^ Pearson’s chi-squared test

### Factors associated with repeat abortion

Table 3 presents the results of multivariate binary logistic regression analysis for repeat abortion of participants. After adjusting for potential confounding factors, participants’ age, education, occupation, parity, cognition of the adverse health effects of having an abortion, and contraceptive use at the time of conception, and the participants’ sexual partners’ age, education, income per month, and willingness to use contraception were significantly associated with the repeat abortion.

**Table 3:** Factors associated with repeat abortion of participants

Variables^a^	Repeat abortion (0 = no, 1 = yes)			***P***-value^b^
	β	S.E.	OR (95% CI)	
**Age of participants** (ref = ≤30 years)				
31–35 years	0.31	0.12	1.37 (1.08–1.73)	0.009
≥ 36 years	0.60	0.18	1.82 (1.29–2.57)	0.001
**Education of participants** (ref = bachelor or above)				
≤ Senior high school	0.62	0.14	1.86 (1.42–2.43)	< 0.001
Junior college	0.38	0.12	1.46 (1.17–1.83)	0.001
**Occupation of participants** (ref = student)				
Jobless	0.90	0.38	2.46 (1.18–5.13)	0.017
**Parity of participants** (ref = no children)				
One child	0.43	0.17	1.54 (1.10–2.17)	0.013
≥ Two children	0.34	0.19	1.40 (0.96–2.03)	0.077
**Cognition of adverse health effects of having an abortion of participants** (ref = know well)				
General	0.47	0.11	1.60 (1.28–1.98)	< 0.001
Don’t know	0.92	0.11	2.51 (2.02–3.11)	< 0.001
**Age of participants’ sexual partners** (ref = ≤30 years)				
31–35 years	0.29	0.12	1.33 (1.06–1.68)	0.015
≥ 36 years	0.76	0.16	2.13 (1.56–2.91)	< 0.001
**Education of participants’ sexual partners** (ref = bachelor or above)				
≤ Senior high school	0.50	0.13	1.66 (1.28–2.15)	< 0.001
Junior college	0.32	0.12	1.38 (1.10–1.74)	0.004
**Income per month of participants’ sexual partners** (ref = ≤4500 Yuan)				
4501–6000 Yuan	0.08	0.10	1.08 (0.89–1.31)	0.450
≥ 6001 Yuan	0.29	0.11	1.34 (1.08–1.65)	0.007
**Willingness to use contraception of participants’ sexual partners** (ref = very strong)				
Strong	0.14	0.09	1.14 (0.95–1.37)	0.148
General	0.17	0.12	1.19 (0.94–1.50)	0.145
Weak or very weak	1.92	0.53	6.84 (2.42–19.33)	< 0.001
**Contraceptive use at the time of conception** (ref = nonuse)				
Condom	0.28	0.10	1.33 (1.09–1.61)	0.004
Rhythm	0.12	0.13	1.12 (0.88–1.44)	0.360
Withdrawal	0.36	0.13	1.43 (1.12–1.84)	0.005
Emergency	0.39	0.15	1.48 (1.09–1.99)	0.011
Other	0.13	0.26	1.14 (0.68–1.91)	0.615

^a^Independent variables in the logistic regression model included participants’ age, education, residence status, migrant status, occupation, marital status, parity, and cognition of possible adverse health effects of having an abortion, participants’ sexual partners’ age, education, occupation, income per month, and willingness to use contraception, and contraceptive use at the time of conception. However, considering the table size, here we just reported the results for these significant variables

^b^ Model fit information: *p*-value of omnibus tests of model coefficients < 0.001, −2LL = 4048.481, Cox & Snell *R*^2^ = 0.147, Nagelkerke *R*^2^ = 0.197, *p*-value of Hosmer-Lemeshow goodness-of-fit test = 0.152

## Discussion

From the global public health perspective, repeat abortion remains a severe challenge to women’s reproductive health, which needs increased attention and research. This study provides relevant evidence from northwestern China to the growing body of international literature on the repeat abortion of women and associated factors from both the perspectives of women and their sexual partners.

Our study found that approximately six in ten (56.6%) participants seeking an abortion with unintended pregnancies were undergoing a repeat abortion, which was similar to previous studies in China that reported that the prevalence of repeat abortion among women ranged from 43.0 to 65.2% [45, 49, 52]. However, these findings about the prevalence of repeat abortion among Chinese women were much higher than that of female abortion seekers elsewhere in the world [4–11, 13–18, 20–28]. No direct evidence has been found about why Chinese women have a higher prevalence of repeat abortion; however, this difference might be relevant to the gap in sexual education and contraceptive practice in China compared with other, especially developed, countries [48]. To some extent, these results in China may also be related to the universal two-child policy implemented since Jan. 1st, 2016. In summary, our findings reveal the seriousness of this reproductive health problem for women in China, and more attention and action should be taken on how to reduce the risks of unintended pregnancy and repeat abortion among Chinese women.

In terms of contraceptive use at the time of conception, 58.3% had used contraception measures and 41.7% were nonuse of contraception, which was consistent with that in previous studies in China [45, 53]. Meanwhile, we found that a significantly higher percentage (61.2%) of women undergoing a repeat abortion had used contraception measures at the time of conception than those receiving their first abortions (54.4%). The results of multivariate analysis further showed that participants who had used contraception measures at the time of conception, such as condom, withdrawal, and emergency contraception, were 1.33–1.48 times more likely to undergo a repeat abortion than those who did not use contraceptives. These findings have also been reported in previous studies [11, 40, 41, 54]; however, McCall et al. [8] and Thapa et al. [25] did not identify a significant association between contraceptive use at the time of conception and repeat abortion among women in Scotland and Nepal, respectively. In addition, although we did not find a significant association of contraceptive use during the 6 months preceding the survey with repeat abortion of women, a slightly higher percentage of women undergoing a repeat abortion had used at least one contraception measure than those receiving their first abortions in this study. As noted by Kabiru et al. [40] and Cohen [54], these results all cast doubt on the often-made assumption that some women rely on abortions as a means to prevent unintended pregnancies and unplanned births, and women having experienced an abortion and even a repeat abortion are less motivated to use contraception. Instead, women having had a previous abortion might be more likely to use contraception but may need counseling for correct and effective contraceptive use and access to a wider range of effective contraception measures, such as the long-acting measures, to minimize the risks of contraceptive failure [40, 45, 46, 54].

Besides, with respect to the participants themselves, their age, education, occupation, parity, and cognition of the possible adverse health effects of having an abortion were significantly associated with the repeat abortion. First, in line with prior studies [4–7, 10–13, 19, 23–26, 28, 41, 44, 49, 52], we found that an increased age of women was strongly associated with a higher risk of having repeat abortions. This association is not surprising and reflects the longer exposure to sexual intercourse and thereby increased risks of unintended pregnancies for these older women. Second, we found that the lower the level of education women attained, the higher their risks of having a repeat abortion, which was consistent with prior studies [4–6, 9, 11, 12, 16, 18, 19, 21, 23, 26, 27, 39, 40, 43, 53]. One possible explanation is that women with a higher-level education might have higher levels of health literacy, especially contraceptive knowledge, and better contraceptive practice, which allow them to better avoid unintended pregnancies and subsequent abortions. However, a few studies conducted in Ghana [20], Nepal [25], and the Netherlands [13] reported a contrary finding with the positive association between a higher-level education of women and the repeat abortion. Third, consistent with prior studies [11, 40, 43, 46, 48, 49], we found that jobless women were 2.46 times more likely to undergo a repeat abortion. This finding might be related to their poor contraceptive knowledge and limited access to contraceptive measures [48]. In addition, Makenzius et al. [43] pointed out that women suffering from poverty caused by unemployment might have reduced motivation to practice safe sexual intercourse.

Parity was the fourth factor associated with repeat abortion. In line with a great deal of evidence in prior studies [4, 5, 8, 9, 11–15, 19, 21, 24–26, 39, 41, 43, 44, 46, 48, 49], our study showed that participants having a child were 1.54 times more likely to under a repeat abortion than nulliparous participants. Jones et al. reported that women having children were demonstrably fertile and therefore at continued risk of pregnancy after the first abortion [4]. In the opinion of Kirkman et al. [55], parous women, especially those with higher parity, sought abortions because they did not want to look after another child. In our study, the association between parity and repeat abortion might also be related to the change in the family planning policy in China. To some extent, the implementation of the universal two-child policy in China since 2016 might weaken the contraceptive awareness of women, especially those having already had one child, as they could legally have a second child, even if it might be an unwanted birth. Fifth, we found that participants’ cognition of potential adverse health effects of having an abortion was significantly associated with repeat abortion. Not surprisingly, we found that the lower the cognitive level among women, the higher the risk of having a repeat abortion. This finding may reflect the weak sex education system in China, and women still have limited access to counseling for relevant reproductive health knowledge; however, no related evidence has been reported in prior studies.

In addition to the above factors, our study identified four more factors that were significantly associated with the repeat abortion from the perspective of abortion seekers’ sexual partners, including their age, education, income, and attitudes toward contraceptive use. Few similar studies and findings have been reported. First, we found that the participants whose sexual partners were older than 30 years were 1.33–2.13 times more likely to undergo a repeat abortion. This is similar to the finding on women’s age as a factor associated with the repeat abortion, and it reflects the longer exposure to sexual intercourse of older sexual partners and thereby higher risks of unintended pregnancies for women. Second, our study reported that abortion seekers whose sexual partners attained a lower-level education were 1.38–1.66 times more likely to undergo a repeat abortion. This is also similar to that of the participants themselves, and their sexual partners with a lower-level education might have less contraceptive knowledge and poorer contraceptive practice. Zhang et al. found that women with a repeat abortion had a higher percentage of sexual partners with a low- and middle-level education compared to those who experienced only one abortion; however, they did not identify a significant association between them [52].

Besides, participants whose sexual partners had the highest-level income per month were 1.34 times more likely to undergo a repeat abortion than those with the lowest-level income per month. One possible explanation is that, in the current structure and relationship of Chinese couples and families, it is still men who carry the most of the economic responsibility; and for these couples or families where men receive a high-level income, they may have the financial ability to take care of a child whose birth is unplanned [40], thereby they are at a higher risk of poor contraception practices. Fourth, our study identified that participants’ sexual partners’ attitudes toward contraception was strongly associated with participants’ repeat abortions. Compared with participants whose sexual partners presented a very strong willingness to use contraception, those with a sexual partner who had a weak or very weak willingness were 6.84 times more likely to undergo a repeat abortion. As we found that the male condom was the most common method participants used at the time of conception or during the 6 months preceding the survey, the negative attitudes toward contraceptive use by sexual partners would not surprisingly increase risk of unintended pregnancy for women. Although no similar evidence about the significant association has ever been reported, Zhang et al. also found that compared with women undergoing a first abortion, those with a repeat abortion had a higher percentage of sexual partners who had negative attitudes toward contraception [52]. These findings highlight the significant role of women’s sexual partners in reducing women’s risks of unintended pregnancy and subsequent abortion, even the repeat abortion, by improving their awareness and practice of contraception.

There are several limitations to our study. First, as the study was conducted in Xi’an, a northwestern city in China, the results could not be generalized very well to all women in other regions in China. Second, although our study focused on both the perspectives of participants and their sexual partners, we could not identify and report all factors associated with the repeat abortion, as there were many other aspects that we did not collect and review. Third, as the survey was self-reported by women seeking abortions themselves, though anonymous, this might bring a bias of social desirability. For example, women who were undergoing a repeat abortion might especially feel like that they should report having used contraception. Fourth, as this was based on a cross-sectional survey, we could not conclude any causal relationships of repeat abortion of women with the factors identified in our study. Fifth, the use of convenience sampling, rather than probability sampling, is a weakness of the study.

## Conclusions

This study found that repeat abortion among abortion seekers is highly prevalent in Xi’an, China. Approximately six in ten women seeking an abortion were undergoing a repeat abortion, which suggests the still critical issue of reducing the risks of unintended pregnancy and repeat abortion among women and improving their reproductive health in China. In addition, this study identified ten factors associated with the repeat abortion from both the perspectives of the participants themselves and their sexual partners. Specifically, women who were more likely to undergo a repeat abortion were those who were older, received a low-level education, were jobless, had a child, had a low-level cognition of possible adverse health effects from abortions, and had used contraception at the time of conception, and women whose sexual partners were older, attained a low-level education, received a high-level income per month, and had a weak willingness to use contraception were more likely to undergo a repeat abortion. The occurrence of repeat abortion could be reduced by offering more access to reproductive health and contraception knowledge to women and their sexual partners and by promoting their correct, consistent, and effective contraception practice.

## Supplementary Information

**Additional file 1.** List and characteristics of medical institutions in the survey.

**Additional file 2.** Questionnaire for women seeking abortion services in Xi’an, China.

**Additional file 3.** Sociodemographic characteristics of participants’ sexual partners.

## Data Availability

Data are available upon reasonable request from the corresponding author.

## Abbreviations

95% CI: 95% confidence interval
IQR: Interquartile range
K-S test: Kolmogorov-Smirnov test
OR: Odds ratio
S.E.: Standard error
STROBE: Strengthening the reporting of observational studies in epidemiology
U.K.: United Kingdom
U.S.: United States
