# Supplementary material for: Repeat abortion and associated factors among women seeking abortion services in northwestern China: a cross-sectional study
Source: BMC Public Health. 2021 Sep 6;21:1626. doi: 10.1186/s12889-021-11653-4 (PMC8422724; doi:10.1186/s12889-021-11653-4)
Supplement: Supplementary file 1 — Additional file 1. List and characteristics of medical institutions in the survey. [file 12889_2021_11653_MOESM1_ESM.docx]

**Table 1** List of 90 medical institutions involved in the survey

| **Medical institutions (*N*=90)** | **Categorization** |
| --- | --- |
| Xijing Hospital | Tertiary, Public, & General |
| The Second Affiliated Hospital of Xi'an Jiaotong University | Tertiary, Public, & General |
| Xi'an Fourth Hospital | Tertiary, Public, & General |
| Xi'an Central Hospital | Tertiary, Public, & General |
| Shaanxi Provincial Second People's Hospital | Tertiary, Public, & General |
| The Fourth People's Hospital of Shaanxi | Tertiary, Public, & General |
| Xi'an North Hospital | Secondary, Public, & General |
| Xi'an Huashan Central Hospital | Secondary, Public, & General |
| Xi'an Dongfang Hospital | Secondary, Public, & General |
| Xi'an Electric Power Central Hospital | Secondary, Public, & General |
| PLA 63750 Military Hospital | Secondary, Public, & General |
| Shaanxi Provincial People's Hospital | Tertiary, Public, & General |
| Xi'an No.1 Hospital | Tertiary, Public, & General |
| Ninth Hospital of Xi'an | Tertiary, Public, & General |
| PLA 323 Hospital | Tertiary, Public, & General |
| PLA 451 Hospital | Tertiary, Public, & General |
| Shaanxi Friendship Hospital | Tertiary, Public, & General |
| Shaanxi Traffic Hospital | Secondary, Public, & General |
| Xi'an Hospital of China Railway First Group Co., Ltd | Secondary, Public, & General |
| Xi'an New Changan Maternity Hospital | Tertiary, Private, & Specialized |
| Xi'an Ciai Maternity Hospital | Secondary, Private, & Specialized |
| The First Affiliated Hospital of Xi'an Medical | Tertiary, Public, & General |
| Xi'an Fifth Hospital | Tertiary, Public, & General |
| Xi'an XD Group Hospital | Tertiary, Public, & General |
| Xi'an Daxing Hospital | Tertiary, Private, & General |
| Angel Women and Children's Hospital of Xi'an | Tertiary, Private, & Specialized |
| Xi'an Hospital of AVIC | Secondary, Public, & General |
| Shaanxi Mineral Hospital | Secondary, Public, & General |
| Tangdu Hospital | Tertiary, Public, & General |
| The Second Affiliated Hospital of Xi'an Medical | Tertiary, Public, & General |
| Shaanxi Hangtian Hospital | Secondary, Public, & General |
| Xi'an Xinghua Hospital | Secondary, Public, & General |
| Xi'an No.3 Hospital | Tertiary, Public, & General |
| Changan hospital | Tertiary, Private, & General |
| Xi'an Hancheng Maternity Hospital | Tertiary, Private, & Specialized |
| Xi'an Xihang Hospital of Genertec Universal Medical Group Co. Ltd. | Secondary, Public, & General |
| Xi'an Beihuan Hospital | Secondary, Public, & General |
| Xi'an Fengcheng Hospital | Secondary, Private, & General |
| Xi'an Chengbei Hospital | Secondary, Private, & General |
| Xi'an Tangcheng Hospital | Secondary, Private, & General |
| Xi'an Zhengtai Hospital | Secondary, Private, & General |
| Yihejia Women and Children's Hospital | Secondary, Private, & General |
| Xi'an Kangle Hospital | Secondary, Private, & General |
| The First Affiliated Hospital of Xi'an Jiaotong University | Tertiary, Public, & General |
| Northwest Women and Children's Hospital | Tertiary, Public, & Specialized |
| Shaanxi Armed Police Corps Hospital | Tertiary, Public, & General |
| North Industries 521 Hospital | Tertiary, Public, & General |
| Xi'an Aerospace General Hospital | Secondary, Public, & General |
| Shaanxi Kangfu Hospital | Secondary, Public, & General |
| Xi'an Qujiang Maternity Hospital | Secondary, Private, & Specialized |
| Xi'an Yanta Tianyou Children's Hospital | Secondary, Private, & Specialized |
| Xi'an Yanta Nengkang Hospital of Integrated Traditional Chinese and Western Medicine | Secondary, Private, & General |
| Xi'an Yanta Women's Hospital | Secondary, Private, & General |
| Xi'an Rihua Hospital | Primary, Public, & General |
| Xi'an Yanliang People's Hospital | Secondary, Public, & General |
| Xi'an 141 Hospital | Secondary, Public, & General |
| Xi'an Yanliang Railway Hospital | Secondary, Public, & General |
| Xi'an 630 Hospital | Secondary, Public, & General |
| Xi'an Lintong People's Hospital | Secondary, Public, & General |
| Xi'an Lintong Maternity and Child Healthcare Hospital | Secondary, Public, & General |
| Shaanxi Nuclear Industry 417 Hospital | Secondary, Public, & General |
| Xi'an Railway Engineering Hospital | Secondary, Public, & General |
| Xi'an Changan District Hospital | Secondary, Public, & General |
| Xi'an Changan District Maternity and Child Healthcare Hospital | Secondary, Public, & General |
| Xi'an Trade Union Hospital | Secondary, Public, & Specialized |
| Xi'an Modern Maternity Hospital | Secondary, Private, & Specialized |
| Xi'an Changan Daqian Hospital | Primary, Private, & General |
| Xi'an Gaoling District Hospital | Secondary, Public, & General |
| Xi'an Gaoling District Maternity and Child Healthcare Hospital | Secondary, Public, & General |
| Gem Flower Xi'an Changqing Staff Hospital | Secondary, Public, & General |
| Xi'an Huyi District Maternity and Child Healthcare Hospital | Secondary, Public, & General |
| Shaanxi Sengong Hospital | Secondary, Public, & General |
| Xi'an Huyi People's Hospital | Secondary, Public, & General |
| Xi'an Huyi Traditional Chinese Medicine Hospital | Secondary, Public, & General |
| Xi'an Huian Hospital | Secondary, Public, & General |
| Xi'an Jiren Hospital | Secondary, Private, & General |
| Xi'an Lantian County People's Hospital | Secondary, Public, & General |
| Xi'an Lantian County Traditional Chinese Medicine Hospital | Secondary, Public, & General |
| Xi'an Lantian County Maternity and Child Healthcare Hospital | Secondary, Public, & General |
| Xi'an Zhouzhi County People's Hospital | Secondary, Public, & General |
| Xi'an Zhouzhi County Traditional Chinese Medicine Hospital | Secondary, Public, & General |
| Xi'an Zhouzhi County Maternity and Child Healthcare Hospital | Secondary, Public, & General |
| Xi'an Zhouzhi United Hospital | Secondary, Private, & General |
| Xi'an Zhongbojixian Hospital | Secondary, Private, & General |
| Xi'an Gaoxin Hospital | Tertiary, Private, & General |
| Affiliated Hospital of Xi'an Medical College | Tertiary, Private, & General |
| Xi'an Liren Hospital | Primary, Private, & General |
| Xi'an Jianqiao Hospital | Primary, Private, & General |
| Xi'an Beiche Hospital | Secondary, Public, & General |
| Jingyang Yongan Hospital | Secondary, Private, & General |

**Table 2** Characteristics of medical institutions that participants attended (*n, %*)

| **Characteristics** | **All abortion seekers (*n=3814*)** | | | | |
| --- | --- | --- | --- | --- | --- |
|  |  | **Abortion seekers with unintended pregnancie*s (n=3397)*** | | | |
|  |  |  | **First abortion (*n=1473*)** | **Repeat abortion (*n=1924*)** | ***P*-value**^†^ |
| **Categorization of level** | | | | |  |
| Tertiary hospital | 2209 (57.9) | 1959 (57.7) | 885 (60.1) | 1074 (55.8) | 0.044 |
| Secondary hospital | 1427 (37.4) | 1286 (37.9) | 527 (35.8) | 759 (39.4) |  |
| Primary hospital | 178 (4.7) | 152 (4.5) | 61 (4.1) | 91 (4.7) |  |
| **Categorization of ownership** | | | | | 0.828 |
| Public hospital | 3052 (80.0) | 2722 (80.1) | 1183 (80.3) | 1539 (80.0) |  |
| Private hospital | 762 (20.0) | 675 (19.9) | 290 (19.7) | 385 (20.0) |  |
| **Categorization of function** | | | | | 0.933 |
| General hospital | 2999 (78.6) | 2677 (78.8) | 1162 (78.9) | 1515 (78.7) |  |
| Specialized hospital | 815 (21.4) | 720 (21.2) | 311 (21.1) | 409 (21.3) |  |

^†^ Pearson’s chi-squared test.
