# Supplementary material for: Repeat abortion and associated factors among women seeking abortion services in northwestern China: a cross-sectional study
Source: BMC Public Health. 2021 Sep 6;21:1626. doi: 10.1186/s12889-021-11653-4 (PMC8422724; doi:10.1186/s12889-021-11653-4)
Supplement: Supplementary file 2 — Additional file 2. Questionnaire for women seeking abortion services in Xi’an, China. [file 12889_2021_11653_MOESM2_ESM.docx]

**Questionnaire for women seeking abortion services in Xi’an, China**

*Dear patients, thank you very much for your participation in this survey. The questionnaire is anonymous. For the questions as below, please choose one selection only, unless otherwise specified.*

| ***Section 1: Sociodemographic characteristics of participants***  **1.1** Age: _____ years old  **1.2** Education: _____  (1) Senior high school or below; (2) Junior college; (3) Bachelor’s degree or above  **1.3** Residence status: _____  (1) Rural resident; (2) Urban resident  **1.4** Migrant status: _____  (1) Migrant; (2) Nonmigrant  **1.5** Occupation: _____  (1) Student; (2) Housework; (3) Farmer; (4) Self-employed;  (5) Enterprise employee; (6) Civil servant or teacher or researcher; (7) Jobless  **1.6** Income per month: _____Yuan  **1.7** Marital status: _____  (1) Unmarried; (2) Married  **1.8** Parity: _____  (1) No children; (2) 1 child; (3) ≥2 children  ***Section 2: Sociodemographic characteristics of participants’ sexual partners***  **2.1** Age: _____ years old  **2.2** Education: _____  (1) Senior high school or below; (2) Junior college; (3) Bachelor’s degree or above  **2.3** Residence status: _____  (1) Rural resident; (2) Urban resident  **2.4** Migrant status: _____  (1) Migrant; (2) Nonmigrant  **2.5** Occupation: _____  (1) Student; (2) Housework; (3) Farmer; (4) Self-employed;  (5) Enterprise employee; (6) Civil servant or teacher or researcher; (7) Jobless  **2.6** Income per month: _____Yuan  ***Section 3: Induced abortion and contraceptive use***  **3.1** Is this your first time to seek an abortion? _____  (1) Yes; (2) No  **3.2** Reason for your current induced abortion? _____  (1) Unintended pregnancy (*go to Q3.3*);  (2) Intended pregnancy but due to medical reasons (*go to Q3.5*)  **3.3** Reason for your current unintended pregnancy? _____  (1) Nonuse of contraception (*go to Q3.5*);  (2) Ineffective contraception (*go to Q3.4*)  **3.4** Contraceptive use for current unintended pregnancy? _____  (1) Condom; (2) Rhythm; (3) Withdrawal; (4) Emergency; (5) Other  **3.5** Contraceptive use during six months preceding the survey? _____(*available for multiple choices*)  (1) Nonuse; (2) Condom; (3) Rhythm; (4) Withdrawal; (5) Emergency; (6) Other  **3.6** Cognition of the possible adverse health effects of having an abortion?_____ (1) Know well; (2) General; (3) Don’t know  **3.7** Your sexual partners’ willingness to use contraception?_____  (1) Very strong; (2) Strong; (3) General; (4) Weak; (5) Very weak |
| --- |
