# Supplementary material for: Repeat abortion and associated factors among women seeking abortion services in northwestern China: a cross-sectional study
Source: BMC Public Health. 2021 Sep 6;21:1626. doi: 10.1186/s12889-021-11653-4 (PMC8422724; doi:10.1186/s12889-021-11653-4)
Supplement: Supplementary file 3 — Additional file 3. Sociodemographic characteristics of participants’ sexual partners. [file 12889_2021_11653_MOESM3_ESM.docx]

**Table 1** Sociodemographic characteristics of participants’ sexual partners

| **Characteristics** | ***N* (%) or Median (IQR)** | | | ***P*-value** |
| --- | --- | --- | --- | --- |
|  | **Total** | **First abortion** | **Repeat abortion** |  |
| **Age** (years) | 32 (28–36) | 30 (26–34) | 33 (30–38) | <0.001^†^ |
| ≤30 years | 1441 (42.5) | 824 (56.1) | 617 (32.1) | <0.001^‡^ |
| 31–35 years | 988 (29.1) | 399 (27.2) | 589 (30.6) |  |
| ≥36 years | 964 (28.4) | 246 (16.7) | 718 (37.3) |  |
| **Education** |  |  |  | <0.001^‡^ |
| ≤Senior high school | 1590 (46.8) | 593 (40.3) | 997 (51.8) |  |
| Junior college | 935 (27.5) | 424 (28.8) | 511 (26.6) |  |
| ≥Bachelor’s degree | 872 (25.7) | 456 (31.0) | 416 (21.6) |  |
| **Residence status** |  |  |  | 0.166^‡^ |
| Rural | 2198 (64.7) | 967 (65.6) | 1231 (64.0) |  |
| Urban | 1199 (35.3) | 506 (34.4) | 693 (36.0) |  |
| **Migrant status** |  |  |  | 0.227^‡^ |
| Migrant | 1218 (35.9) | 539 (36.6) | 679 (35.3) |  |
| Nonmigrant | 2179 (64.1) | 934 (63.4) | 1245 (64.7) |  |
| **Occupation** |  |  |  | <0.001^‡^ |
| Student | 59 (1.7) | 47 (3.2) | 12 (0.6) |  |
| Housework | 41 (1.2) | 20 (1.4) | 21 (1.1) |  |
| Farmer | 293 (8.6) | 111 (7.6) | 182 (9.5) |  |
| Self-employed | 1110 (32.7) | 396 (27.0) | 714 (37.1) |  |
| Enterprise employee | 1356 (40.0) | 635 (43.2) | 721 (37.5) |  |
| Civil servant etc. | 473 (13.9) | 229 (15.6) | 244 (12.7) |  |
| Jobless | 60 (1.8) | 31 (2.1) | 29 (1.5) |  |
| **Monthly income** (Yuan) | 5000 (4000–8000) | 5000 (4000–8000) | 5000 (4000–8000) | 0.004^†^ |
| ≤4500 Yuan | 990 (29.4) | 460 (31.5) | 530 (27.8) | 0.016^‡^ |
| 4501–6000 Yuan | 1166 (34.7) | 511 (35.0) | 655 (34.4) |  |
| ≥6001 Yuan | 1208 (35.9) | 488 (33.4) | 720 (37.8) |  |

^†^ Two-sample K-S test. ^‡^ Pearson’s chi-squared test.
